# Mechanical and Computational Fluid Dynamic Models for Magnesium-Based Implants

**DOI:** 10.3390/ma17040830

**Published:** 2024-02-08

**Authors:** Veronica Manescu (Paltanea), Gheorghe Paltanea, Aurora Antoniac, Lucian Gheorghe Gruionu, Alina Robu, Marius Vasilescu, Stefan Alexandru Laptoiu, Ana Iulia Bita, Georgiana Maria Popa, Andreea Liliana Cocosila, Vlad Silviu, Anca Porumb

**Affiliations:** 1 Faculty of Material Science and Engineering, National University of Science and Technology Politehnica Bucharest, 313 Splaiul Independentei, District 6, RO-060042 Bucharest, Romania; veronica.paltanea@upb.ro (V.M.); alinarobu2021@gmail.com (A.R.); lucian.vasilescu@upb.ro (M.V.); stef.laptoiu@gmail.com (S.A.L.); 2 Faculty of Electrical Engineering, National University of Science and Technology Politehnica Bucharest, 313 Splaiul Independentei, District 6, RO-060042 Bucharest, Romania; gheorghe.paltanea@upb.ro; 3 Faculty of Mechanics, University of Craiova, 13 Alexandru Ioan Cuza, RO-200585 Craiova, Romania; lgruionu@gmail.com; 4 Department of Surgical Disciplines, Faculty of Medicine and Pharmacy, University of Oradea, 10 P-ta 1 December Street, RO-410073 Oradea, Romania; pascalau.georgiana17@yahoo.com (G.M.P.); andreea_cocosila93@yahoo.com (A.L.C.); dr_silviu_vlad@yahoo.com (V.S.); 5 Department of Dental Medicine, Faculty of Medicine and Pharmacy, University of Oradea, 10 P-ta 1 December Street, RO-410073 Oradea, Romania; anca.porumb@uoradea.ro

**Keywords:** Mg-based implants, 3D printing implants, mechanical properties, finite element analysis, permeability, computational fluid dynamics

## Abstract

Today, mechanical properties and fluid flow dynamic analysis are considered to be two of the most important steps in implant design for bone tissue engineering. The mechanical behavior is characterized by Young’s modulus, which must have a value close to that of the human bone, while from the fluid dynamics point of view, the implant permeability and wall shear stress are two parameters directly linked to cell growth, adhesion, and proliferation. In this study, we proposed two simple geometries with a three-dimensional pore network dedicated to a manufacturing route based on a titanium wire waving procedure used as an intermediary step for Mg-based implant fabrication. Implant deformation under different static loads, von Mises stresses, and safety factors were investigated using finite element analysis. The implant permeability was computed based on Darcy’s law following computational fluid dynamic simulations and, based on the pressure drop, was numerically estimated. It was concluded that both models exhibited a permeability close to the human trabecular bone and reduced wall shear stresses within the biological range. As a general finding, the proposed geometries could be useful in orthopedics for bone defect treatment based on numerical analyses because they mimic the trabecular bone properties.

## 1. Introduction

Lately, one can notice that the continuous development of the tissue engineering domain is growing fast and becoming appealing for researchers investigating biocompatible material manufacture and artificial bone substitutes. It is estimated that man-made grafts will cover an important part of the worldwide tissue demand, so a reduction in the number of autografts and allografts, which are considered today as the “gold standard”, could be foreseen [1,2]. Three-dimensional (3D) porous implants or scaffolds exhibiting various geometries are used to treat patients who need a complex and difficult surgical intervention generated by a hard tissue absence due to an accident, infections of the primary endoprosthesis, or bone oncological pathologies. Schemitsch et al. [3] classified bone defects. They considered small defects as those that present a size lower than 2 cm and a cortical bone circumference loss below 50%. Intermediate bone defects have geometrical dimensions between 2 cm and 6 cm and a circumference loss higher than 50%, and large bone defects with a minimum size of 6 cm are considered critical-size defects. It is well known that such defects cannot spontaneously heal during the patients’ lives. On the other hand, for defects smaller than 2.5 cm, a natural healing process with no scars is observed [4,5]. When a bone substitute is present, the bone healing mechanism consists of new bone formation around the implant, stabilizing its position at the bone defect place. The osteogenesis process strongly depends on the implant’s chemical composition and surface topography [6]. Starting with the moment when the implant is inserted into the patient’s body, it enters into contact with human blood, leading to platelet apparition and activation on the implant surface. This phenomenon simultaneously occurs with an inflammatory process generated by the macrophages and neutrophils that ends with hematoma formation. Thrombocytes and leucocytes create a beneficial medium for mesenchymal stem cell (MSC) recruitment. This type of cell will differentiate into osteoblasts through the contact osteogenesis process. The osteoblasts will later form a layer-by-layer substrate at the bone defect zone along the bone edges [7]. The new bone formation also occurs in the opposite direction, oriented from bone edges to implant, in the framework of a phenomenon known in the literature as distance osteogenesis [6]. Contact and distance osteogenesis are important in new and immature bone formation, filling the free space between the implant and the bone. The bone remodeling process in the peri-implant zone substitutes the immature bone with a mature one, resulting in increased mechanical resistance of the bond between the human bone and implant. This process is continuous and has an important contribution to securing the connections that occur at the bone–implant interface. However, in some cases, implant demobilization and failure are present due to insufficient development of the bone in the peri-implant area caused by a specific disease or the stress shielding effect, which consists of a mismatch between the Young’s modulus value of bone and implant.

Different production techniques for implants were developed based on an additive manufacturing process, which permits obtaining anatomical configurations and using many biocompatible materials [8,9]. In addition, designing an implant with adequate architecture similar to the human bone is a complex procedure. One should consider the pore size, shape, and implant total porosity [10]. The literature research evidenced that the implant porosity must be between 25% and 90%, and the pore size has to be included in the following interval, starting with 10 μm and ending with 1000 μm [11,12,13]. All these parameters exhibit an important influence on the implant’s mechanical properties and biocompatibility. A high porosity must be considered when the implant is populated with cells, and beneficial conditions with properties characteristic for increased cell viability are necessary [14]. Concomitantly with the abovementioned advantage, highly porous structures are associated with decreased mechanical properties. As a direct consequence, implant porosity must be correctly chosen to entirely respect bone’s mechanical properties and the application for which it is designed. The medical performance of an implant depends on material properties, topological requirements, and production parameters. Generally, after the implants are manufactured, they are populated with cells developed and multiplied inside a bioreactor before the implantation step.

The most important mechanical properties of orthopedic implants are the elasticity modulus and the yield strength because these implants are usually used in load-bearing zones [15,16]. The human bone has a Young’s modulus between 0.5 GPa and 20 GPa [17,18]. This parameter is essential and must be considered when an implant dedicated to orthopedics is designed. For high-elasticity modulus values, it can be noticed that the implant porosity is reduced and can be useful for intense mechanical solicitation zones [19]. The beneficial effect of mechanical stimuli against the differentiation and proliferation of living cells inside the implant is well known, so the elasticity modulus can be considered one of the most adequate mechanical properties for cell activity monitoring. Finite element analysis (FEA) is considered one of the most important steps in orthopedic implant design today because its geometry can be controlled and modified without the involvement of expensive and invasive experimental mechanical tests, which are usually based on many samples.

The design method and fabrication parameters are chosen as a function of fluid transport capabilities through the implant because the flow of blood or simulated body fluid used in bioreactors for cell development is essential for oxygen and nutrient delivery and also to eliminate the carbon dioxide and waste through blood circulation [20,21]. The biological behavior strongly depends on the fluid–implant interaction, resulting in the wall shear stress (WSS) that is of utmost importance for the cell’s overall viability. Experimental fluid flow analyses are expensive and characterized by numerous limitations, such as high costs, long observation time, and precise results without fluid flow perturbations. An easy solution consists of computational fluid dynamics (CFD) analysis, which is considered an appropriate way to understand and predict the in vivo performance of an implant. This method permits the implant permeability computation [22,23]. The fluid pressure and the wall shear stress have a major impact on the mechanical stimulation of the cells by influencing the level of nitric oxide, which is an important parameter in the human body [24]. Different studies [24,25,26,27,28] proved that WSS influenced the pluripotent stem cells’ differentiation into osteoblasts, cardiac cells, or endothelial cells. It was noticed that values of WSS below 30 mPa had a positive effect on MSC activity, proliferation, and differentiation. At the same time, for values between 0.11 and 10 mPa, increased differentiation of stem cells into osteogenic cells was evidenced, while for WSS comprising a 0.55–24 mPa interval, an accelerated hard tissue mineralization was noticed [29]. The literature revealed that WSS values higher than 60 mPa are in a direct relationship with cellular death [30,31,32,33,34].

Implant material plays an important role in establishing its mechanical and biological performance. Many investigations reported as adequate for bone tissue engineering various biodegradable materials from different classes, such as polymers (polycaprolactone, polyglycolic acid, polylactic acid) [35,36,37], ceramics (tricalcium phosphate, bioglasses) [38,39,40], and metals (zinc and magnesium-based alloys) [4,41,42,43,44,45].

In the present study, we have chosen a biodegradable commercial Mg-based alloy (Mg-Nd-Y-Zr-Zn) produced by Dead See Magnesium Be’er Sheva, Israel, and traded under the name MRI201S. This paper’s aim consists of designing and analyzing two geometrical configurations, which will be practically manufactured as a future study based on a combination of an additive-manufactured polymeric matrix and titanium wire orthogonal waving procedure. Firstly, we will print the two proposed geometries based on fused deposition modeling technology using biodegradable and biocompatible polymers. Then, inside the matrix pores, we will insert and wave Ti6Al4V (medical Ti Grade 5) wires with different diameters. After that, the polymer matrix will be dissolved based on the immersion of Ti-polymer structures in dedicated solvents. The resulting titanium waving will be introduced in a liquid magnesium alloy, and a biocompatible bi-metallic structure will be obtained. We will eliminate the Ti6Al4V wires by introducing the structure mentioned above in hydrofluoric acid, and the Mg parts will be unaffected [46], so we expect to obtain similar geometries to those analyzed in this paper.

Many studies were conducted in the literature that analyzed the mechanical and fluid flow behaviors of implants with different geometric architectures. They found that Young’s modulus and porosity values are very important and must be considered together when the mechanical properties are analyzed. Hutmacher et al. [10] underlined that designing and manufacturing an ideal scaffold could be very complicated, and many parameters must be considered. They recommended that analyzing composite materials and simple implant geometries is the easiest way to modify and consider adequate bone tissue engineering (BTE) geometries. Boccaccio et al. [14] developed a mechanobiology-based algorithm to optimize BTE implant microstructure. The authors considered mechanical analyses expensive and destructive, and performing an FEA during the first stage of implant design compulsory. They searched for an ideal geometry by considering parameters such as pore shapes, their spatial positions, and the number of pores per unit area. Based on Young’s modulus computation and comparing these values with those of human bone, the authors concluded that the rectangular pore structure was characterized by a more considerable predicted amount of new bone compared to the square pores. In addition, elliptic pores proved to be much more beneficial to cell development than circular ones. It can be noticed that the implant geometry features are very important, and simple geometries are preferable over complicated ones because they are much more practically realizable. Nune et al. [16] chose a mesh structure made of biocompatible Ti alloy that proved to sustain the cell development and viability. The implant showed a Young’s modulus of about 4.36 GPa and a porosity of 70%. Ali and Sen [47] designed gyroid- and lattice-based architecture implants with regulated and commonly used geometries. They performed FEA and CFD analysis by choosing Ti6Al4V as the implant material and water as the fluid. They concluded that the mechanical properties of the implant were in the biological range. Still, regarding the CFD analysis, higher permeability and lower WSS values were obtained for the lattice-based models. Zhao et al. [48] investigated the fluid shear stress in BTE implants with cubical and spherical pore architectures. They provided a mathematical approach that can be followed in experiments to estimate the wall shear stress as a function of pore size, total porosity, and entire architecture. One can also notice that simple and regulated geometries were preferred in this case. The authors concluded that combining the FEA and CFD analysis is of utmost importance in implant design and offered information regarding the biological range for WSS, implant materials, and mechanical testing conditions. Much more complex structures were developed by Li et al. [49], which were based on patient medical images of vertebral cancellous tissue. Although the implant design is much more realistic in this case, precise control, optimization, and modification of the geometrical parameters are hard to obtain, and expensive devices are needed, so as a direct consequence, much more effort must be conducted to achieve adequate mechanical properties and fluid flow behavior in the biological range. Fallah et al. [50] designed and made practical innovative designs of BTE implants. Unfortunately, these structures are very hard to implement for Mg-based scaffolds due to the necessity of expensive printing devices, which are suitable in most cases for the additive manufacturing of polymeric scaffolds. Considering the state of the art in the literature, we designed and analyzed two simple implant geometries that can be practically implemented as described above to validate the proposed design through future experimental testing. In our opinion, our implants can be manufactured through an innovative method that does not involve expensive and complicated devices, and their properties are similar to those produced through additive manufacturing of magnesium. Our method can avoid some dangerous situations that can occur in other cases due to the magnesium powder’s pyrophoric character.

**Figure 1:**
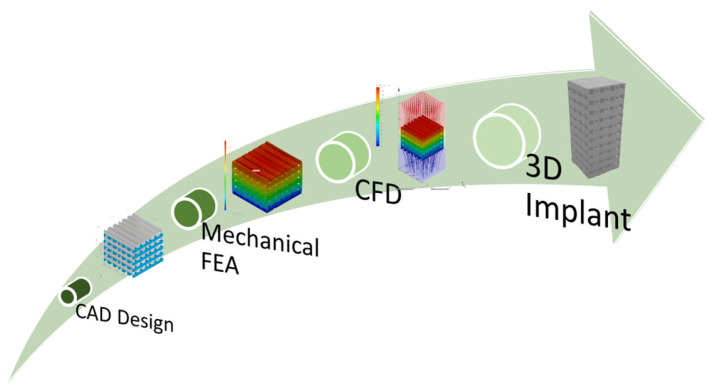
Process diagram comprised of CAD design, mechanical FEA simulation, and CFD analysis for 3D implant manufacturing.

Figure 1 presents a general process diagram that must be followed when 3D implants for bone tissue engineering are designed.

## 2. Modeling and Simulation

### 2.1. Scaffold Design

**Figure 2:**
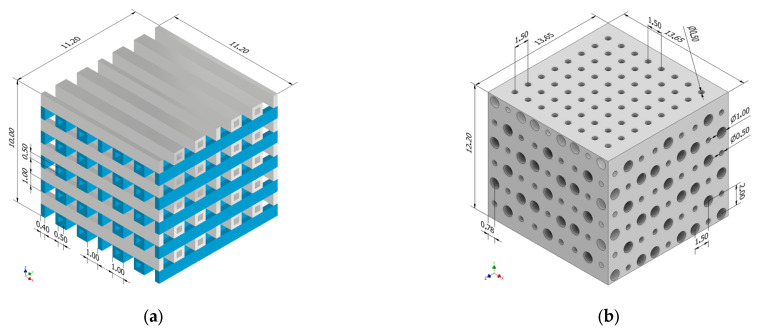
Architecture of the two developed implants: (**a**) PP geometrical configuration; (**b**) PC geometrical configuration.

Computer-aided design (CAD) models were developed using Autodesk Inventor^®^ Professional 2021 mechanical design software. The first geometrical configuration (PP) corresponds to an implant with a base area of 11.20 mm × 11.20 mm, height of 10 mm, and two types of pores with square shapes of the cross-sectional area, having edge dimensions of 1 mm and 0.5 mm, respectively. The second implant exhibited a base area of 13.65 mm × 13.65 mm, a height of 12 mm, and circular-shaped pores with diameters of 1 mm and 0.5 mm. Figure 2 shows the two CAD models developed in the study.

The implants’ porosity *P* was computed based on the following equation:

(1) $$P=\frac{V_{s}-V_{t}}{V_{t}}\cdot 100,$$

where *V_S_* represents the total volume of a cuboid considered in the absence of a pore network, and *V_t_* is the implant volume. The obtained results are presented in Table 1.

**Figure 3:**
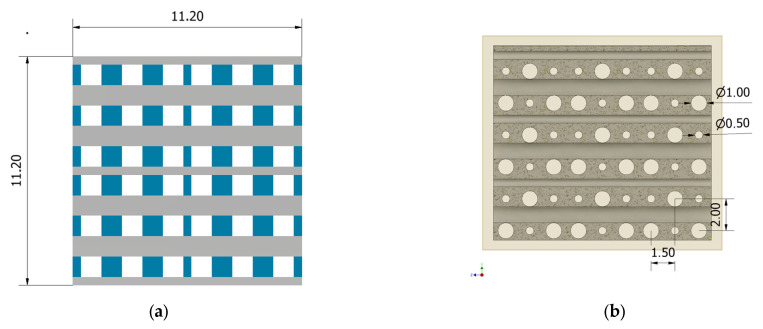
Geometric details of the two developed implants: (**a**) axial view for PP geometry; (**b**) coronal view for PC geometry.

In the case of the CAD model with square-shaped pores, we developed a layered geometry with elements drawn in the XZ and YZ planes, respectively, which were then extruded in Y and X directions with a length of 11.2 mm for each case. Regarding the geometrical configuration based on pores with round-shaped cross-sections, we considered a superposed distribution in YZ and XY planes with an extrusion of 13.65 mm along the X and Z directions. To ensure the possibility of vertical fluid flow inside the implants, circular pores were designed with a diameter of 0.5 mm along the entire structure height. In Figure 3 is presented an axial view of the PP geometry and a coronal view in the case of the PC model.

### 2.2. Mechanical Simulation

In implant design and practical manufacturing, the mechanical properties must be known and well-tuned in accordance with medical applications. Of all these properties, one of the most investigated is Young’s modulus, which must have values close to those of human bone. The analysis of the elastic region was performed using Autodesk Inventor Nastran^®^ 2021 stress analysis software, assuming that the load has a constant value and the dependence between stress and strain is linear. This type of investigation can be considered adequate in the case of medical implants because, inside the human body in non-loading zones, the stress–strain curve evolves in most cases in the linear region.

To compute Young’s modulus, estimate the mechanical stress, and measure the displacement, the two designed CAD models were placed between two rigid bodies with thicknesses of 0.2 mm and areas equal to the base area of the implants. The applied mechanical test was compression in accordance with many literature studies [47,51,52,53,54,55,56]. To model the real behavior of a universal testing machine, we applied a surface-distributed force on the Z axis on the superior rigid body. In contrast, the inferior rigid body was fixed, with all freedom degrees blocked. Regarding the implant material, a Young’s modulus of 44.2 GPa, Poisson ratio of 0.27, compressive yield strength of 190 MPa, and density of 1.79 g/cm^3^ were adopted in good accordance with the mechanical properties of Mg-Nd-Y-Zr-Zn manufactured using the gravity casting method [41,42,57,58,59,60,61,62,63].

**Figure 4:**
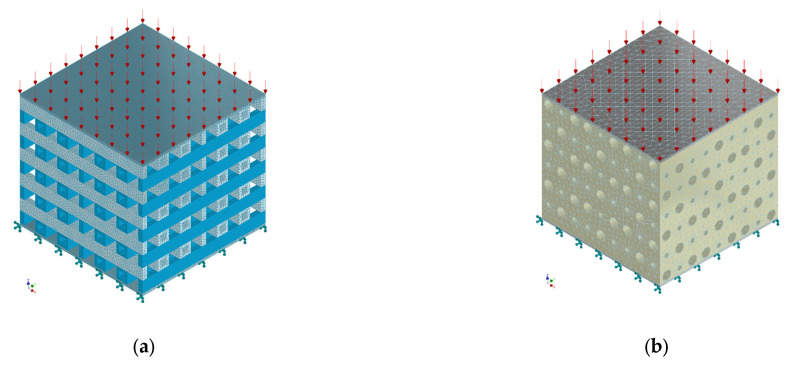
CAD geometries with imposed mechanical load (red arrows) and fixed rigid surface (green fixators): (**a**) PP geometry; (**b**) PC geometry.

We used a mesh with 761,490 nodes and 399,714 tetrahedral second-order elements with a maximum size of 0.2 mm for the PP geometry. In the case of the PC model, the mesh had 646,295 nodes and 401,617 tetrahedral second-order elements with a 0.7 mm maximum size. All the mechanical simulations presented in the Results and Discussion section were performed by keeping constant mesh for both implants, respectively. In Figure 4 are presented the applied mechanical boundary conditions and the mesh for the two CAD models.

### 2.3. Computational Fluid Dynamics Simulation

Computational fluid dynamics represents a simple numerical analysis necessary for investigating the hemodynamic and biological performance of 3D implants characterized by different porosity grades and pore shapes. In the framework of our study, we adopted some idealizations. Firstly, we considered that the fluid has Newtonian behavior by assuming its viscosity has a constant value. This simplification is important because non-Newtonian fluids could have a different effect relative to the wall shear stress that acts on the implant wall and highly influences cell viability [64,65]. Secondly, we adopted different values for the fluid flow velocity. These values were similar to those of blood flow in the case of bone defects located in the long bones of the human body and to those in bioreactors [66,67]. Lastly, the implant surface was supposed to be smooth by considering the wall roughness equal to zero. When the wall roughness is not null, the interactions between implant walls and fluid flows change compared to the ideal case we considered [68].

The numerical CFD simulation was performed with Autodesk^®^ CFD 2021. Based on the computed results, it is possible to determine the implant permeability and the wall shear stress, which are of utmost importance in CFD analysis. By investigating some literature studies, we have chosen the fluid Dullbecco’s Modified Eagle Medium (DMEM) with a density of 1 g/cm^3^ and a viscosity of 0.00145 Pas [51,69]. We assumed that DMEM is uncompressible and has a Newtonian character, and the implants are rigid bodies inside the fluid domain [34,66]. In the idealizations’ case mentioned above, the Navier–Stokes equation (Equation (2)) for incompressible fluids is adequate to solve the problem of mass and momentum conservation along the implants based on the finite volume elements’ method (FVM) [70].

(2) $$\rho \frac{\partial u}{\partial t}-\mu \nabla^{2}u+\rho \left(u\cdot \nabla \right)u+\nabla p=F,u\cdot \nabla =0,$$

where ρ is the fluid density, *u* represents the fluid flow velocity, *t* is the time variable, μ is the fluid viscosity, ∇ is the Del operator, *p* is the fluid pressure, and *F* represents a gravitational or centrifugal force. According to Vossenberg et al. [70] and Xue et al. [71], we imposed *F* = 0. The implant permeability *k* was computed as described by Truscello et al. [72] by taking into account the Darcy relationship:

(3) $$k=\frac{u\mu L}{\left(P_{\max}-P_{\min}\right)},$$

where *L* represents the implant height, *P*_max_ is the maximum pressure determined after the CFD analysis, and *P*_min_ is the minimum pressure in all cases equal to zero. It was noticed that for high values of fluid flow velocity, the Darcy law does not correctly estimate the implant permeability [28,65,67,73]. When the Reynolds number (*R_e_*) is higher than 8.6, the classical Darcy relationship becomes inapplicable [65,67]. To estimate the applicability of Darcy’s law, we computed the Reynolds number following [26,65]:

(4) $$R_{e}=\frac{\rho ud}{\mu },$$

where *d* is the maximum pore size. In our study, because the proposed structure has different pore sizes, we considered in Equation (4) the maximum pore size to be a significative geometrical dimension, as mentioned in [26,65,67].

By considering a laminar flow for the Newtonian fluids, the wall shear stress (WSS τ*_w_*) can be defined to be equal to the first derivative of the fluid flow velocity in the perpendicular direction to the wall, as presented by Eagger et al. [74]:

(5) $$\tau_{w}=\mu \frac{\partial u}{\partial n},$$

where *n* is the normal wall direction.

The boundary conditions for the CFD simulations consisted of an imposed variable velocity value on the inlet surface and null pressure on the outlet surface. The implant walls were set for hydrophilic material behavior and modeled in Autodesk^®^ CFD as implicit “no slip” boundary conditions [72,75,76]. The fluid domain was designed as a cuboid (12.32 mm × 12.32 mm × 30 mm) around the Mg alloy implants. Regarding the Mg-Nd-Y-Zr-Zn, we considered the following values for thermal conductivity 91 W/(mK) in a homogenous distribution along X, Y, and Z axes, specific heat 1050 J/(kg K), and electrical resistivity 6.20 × 10^−8^ Ωm [57,58,59,60,61,62]. The fluid and implant temperature was established at 37 °C to simulate the physiological conditions inside the human body. In addition, we have chosen a shear stress transport turbulence model (SST) of the K-Omega type with an average value of 0.05 for turbulence stated by Bozzi et al. [77], due to the fact that turbulence is a physiological property of the biological fluids and occurs in the case of bone injuries and other pathologies that involve blood flow [78,79,80].

**Figure 5:**
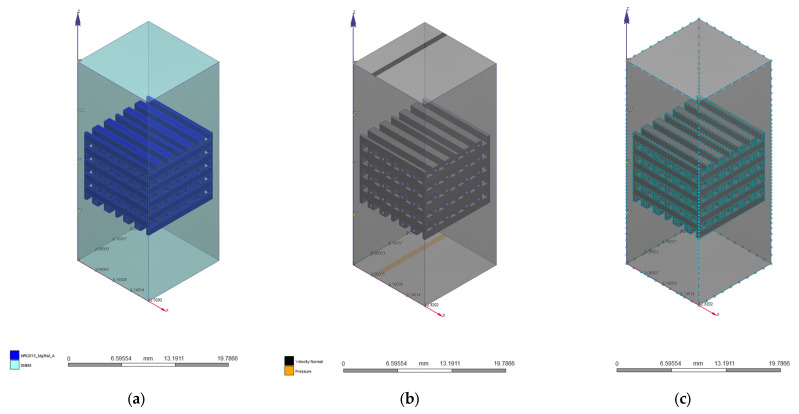
CFD simulation for the PP implant: (**a**) fluid domain; (**b**) boundary conditions; (**c**) mesh with 340,000 volume elements.

**Figure 6:**
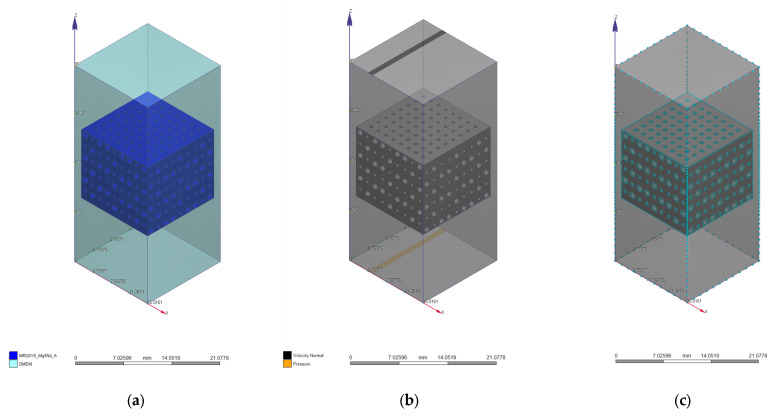
CFD simulation for the PC implant: (**a**) fluid domain; (**b**) boundary conditions; (**c**) mesh with 1,275,053 volume elements.

In Figure 5 and Figure 6 are presented the fluid domain, boundary conditions, and the mesh used for the analyzed problems developed in the case of two CAD models.

## 3. Results and Discussion

### 3.1. Mechanical Simulation Results

The Young’s modulus of the implants was computed based on results obtained from the mechanical simulations. The applied force was variated between 50 N and 1000 N with a step of 100 N. This type of load was chosen by the following criteria: it was supposed that the implants would be placed into a patient’s femur to treat a bone defect, the patient has an average body mass index, and against the leg would be applied a maximum force equal to 10% of body weight. Table 2 presents the results of mechanical simulations for PP and PC geometries used in the elasticity modulus computation (Equation (6) [47]).

(6) $$E_{t}=\frac{FL}{dA},$$

where *F* represents the applied force distributed on the superior rigid body surface, *L* is the implant height, *d* is associated with the maximum displacement value computed with Autodesk Inventor Nastran^®^, and *A* is the cross-sectional area (for PP model 125.44 mm^2^, for PC model 186.33 mm^2^).

**Figure 7:**
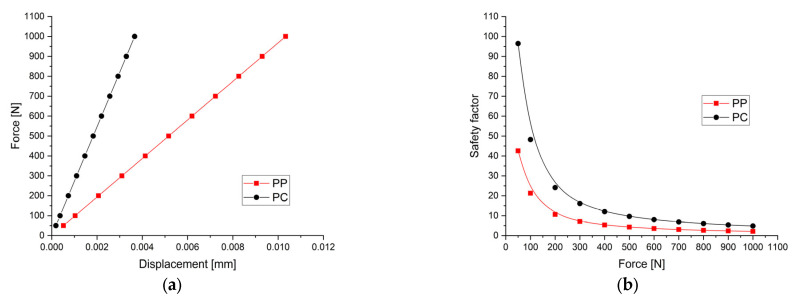
Graphical dependencies obtained after the mechanical simulations made for the two implants: (**a**) force versus displacement variation used in the computation of mechanical rigidity of the implants; (**b**) safety factor dependence on the applied load.

For the PP implant, the theoretical elasticity modulus computed based on Equation (6) was equal to 7.71 GPa, and a very good convergence of the results presented in Table 2 was evidenced. The implant rigidity was estimated at 96,837.38 N/mm and mathematically determined as the slope of force–displacement variation (Figure 7a). The safety factor is an important parameter that is usually used to see if an implant is correctly designed for different applied loads. In the case of a good mechanical resistance, its value must be higher than 1. A Young’s modulus of about 17.88 GPa was obtained for the PC configuration. Using Equation (6) and the data presented in Table 2, it can be noted that all the computed values were equal to the elasticity modulus mentioned above. The implant rigidity was determined as in the other case and was estimated at 273,125 N/mm (Figure 7a). In addition, the safety factor for the PC design exhibited values higher than 4.82 in comparison with 2.12 in the case of the PP model. Both values were obtained for an applied force of 1000 N (Figure 7b).

As a general conclusion, both developed implants have Young’s modulus values between 0.5 and 20 GPa, which are adequate for bone defect treatment in good accordance with the literature [17]. The differences between elasticity modulus are due to the porosity grade and pore shape variation. The PC model can be used in intense load-bearing zones, exhibiting a higher elasticity modulus value. However, this value is lower than the upper limit of 20 GPa determined for the human bone. For the PP model, one can notice an excellent value for Young’s modulus and we suggest using this implant for patients with trabecular bone defects. In both cases, the minimum value of the safety factor is higher than 1, a fact that is linked to good mechanical resistance, so we do not foresee implant failure. Figure 7 presents the variation of force versus maximum implant displacement and the safety factor as a function of the applied load.

**Figure 8:**
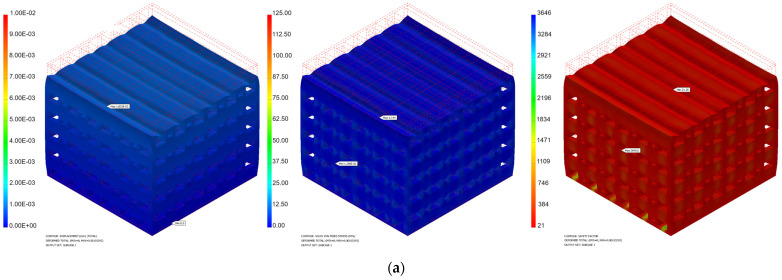

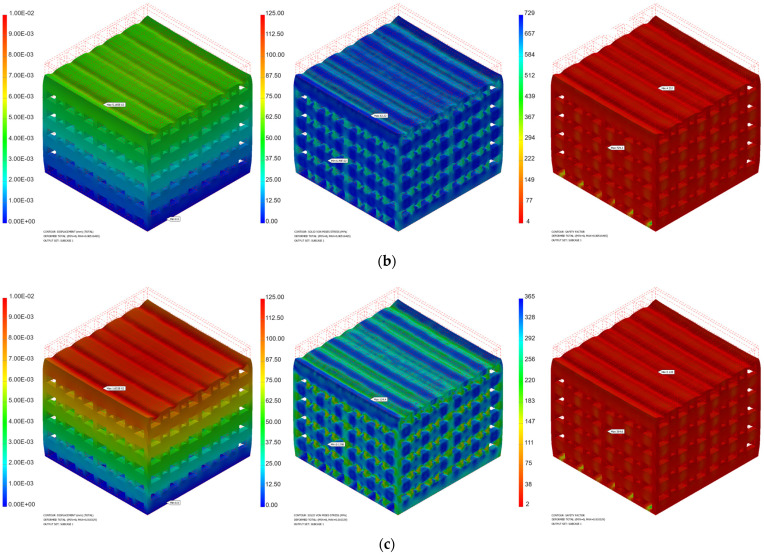
Numerical simulation results obtained for the PP implant (displacement, von Misses stress, safety factor) in the cases of different applied loads: (**a**) 100 N; (**b**) 500 N; (**c**) 1000 N.

**Figure 9:**
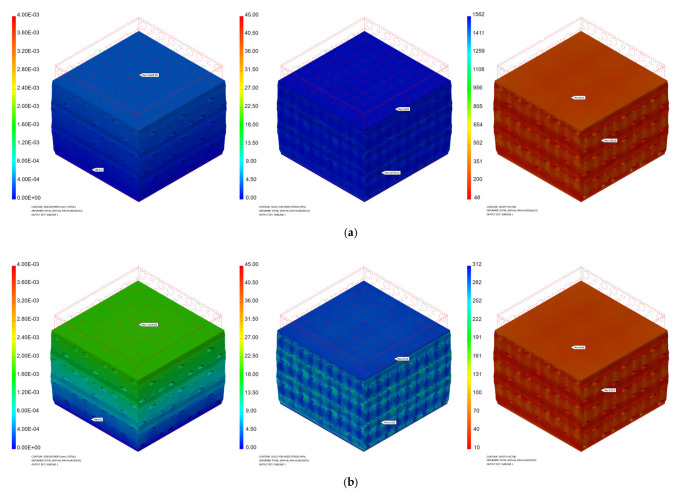

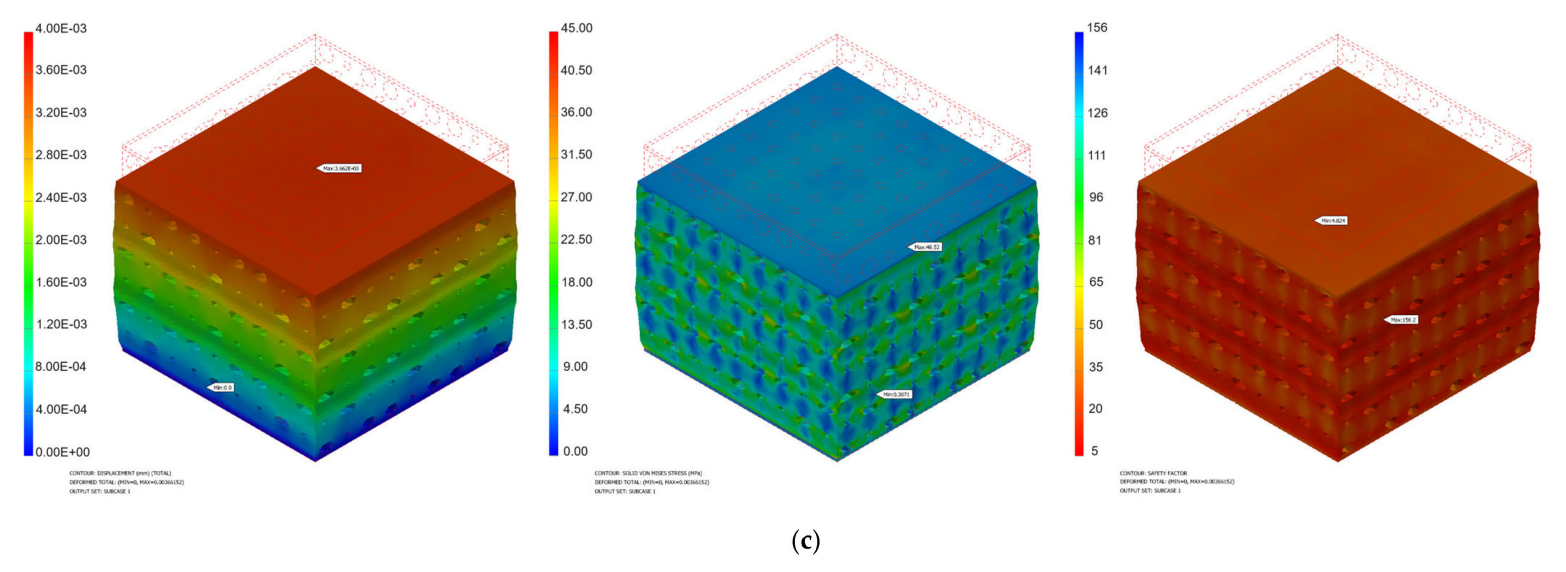
Numerical simulation results obtained for the PC implant (displacement, von Misses stress, safety factor) in the cases of different applied loads: (**a**) 100 N; (**b**) 500 N; (**c**) 1000 N.

Figure 8 and Figure 9 present some numerical results obtained for different values of the applied force.

### 3.2. Computational Fluid Dynamics Simulation Results

**Figure 10:**
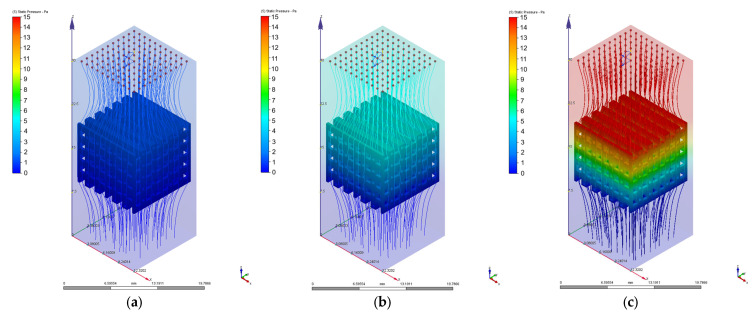
Pressure drops in the case of PP implant for different values of the inlet velocity set as boundary conditions: (**a**) 1 mm/s; (**b**) 4 mm/s; (**c**) 10 mm/s.

**Figure 11:**
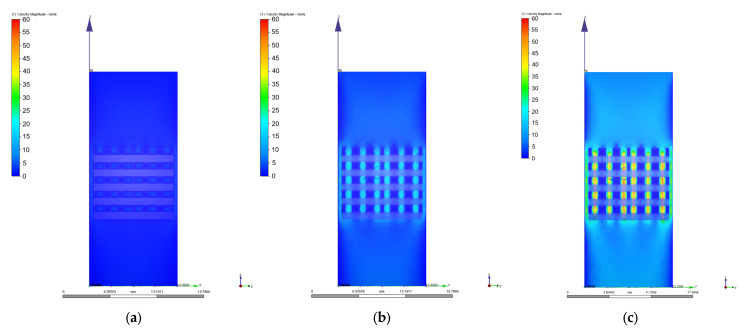
Variation of the fluid flow velocity in the case of PP implant for different values of the inlet velocity set as boundary conditions: (**a**) 1 mm/s; (**b**) 4 mm/s; (**c**) 10 mm/s.

**Figure 12:**
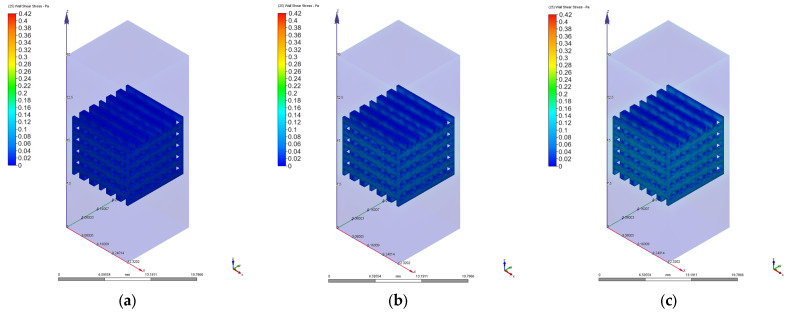

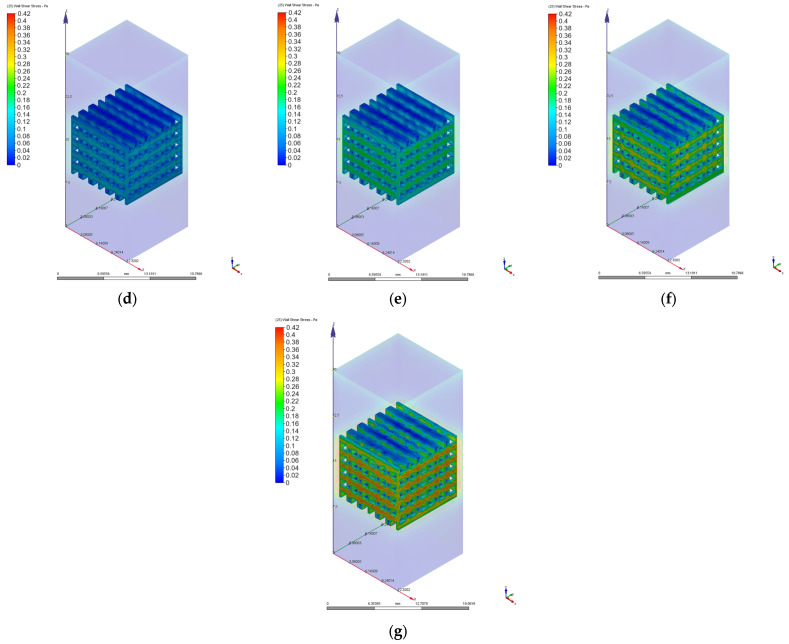
Wall shear stress variation for the PP implant at different values of the inlet velocity set as boundary conditions: (**a**) 1 mm/s; (**b**) 2 mm/s; (**c**) 3 mm/s; (**d**) 4 mm/s; (**e**) 6 mm/s; (**f**) 8 mm/s; (**g**) 10 mm/s.

**Figure 13:**
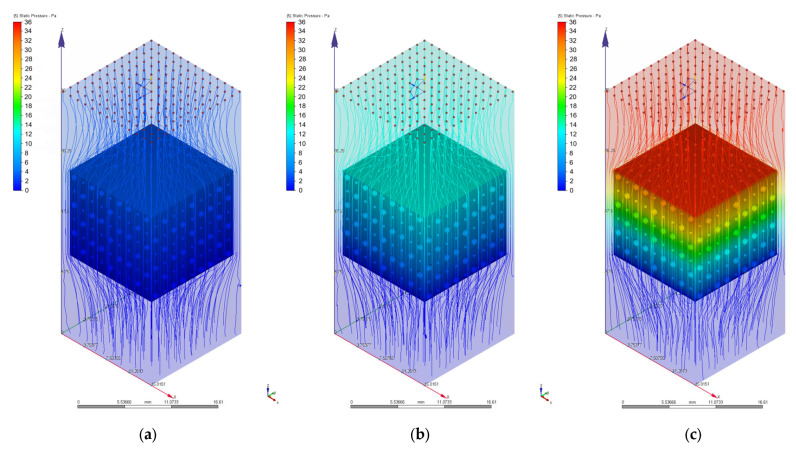
Pressure drops in the case of PC implant for different values of the inlet velocity set as boundary conditions: (**a**) 1 mm/s; (**b**) 4 mm/s; (**c**) 10 mm/s.

**Figure 14:**
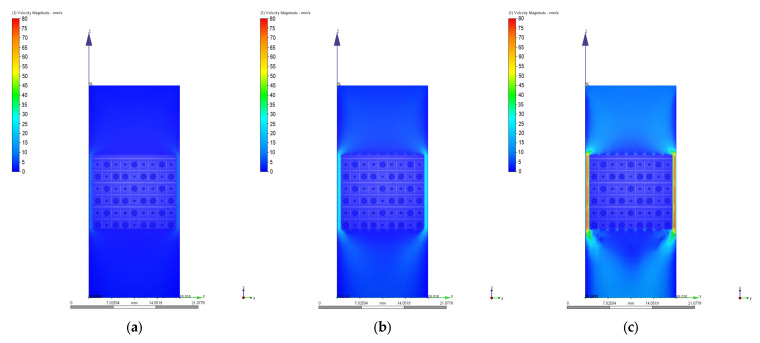
Variation of the fluid flow velocity in the case of PC implant for different values of the inlet velocity set as boundary conditions: (**a**) 1 mm/s; (**b**) 4 mm/s; (**c**) 10 mm/s.

**Figure 15:**
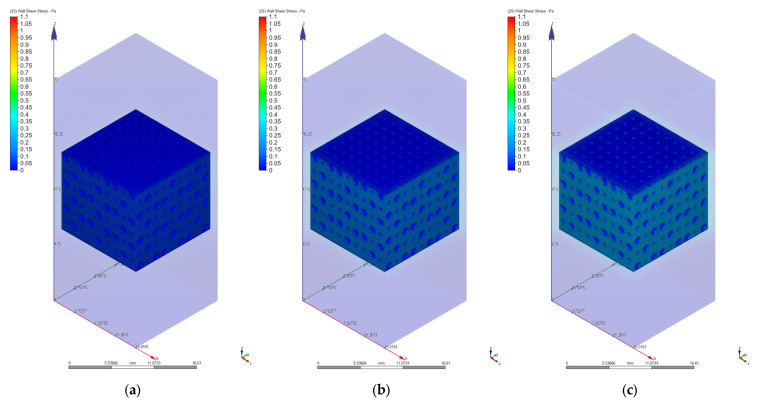

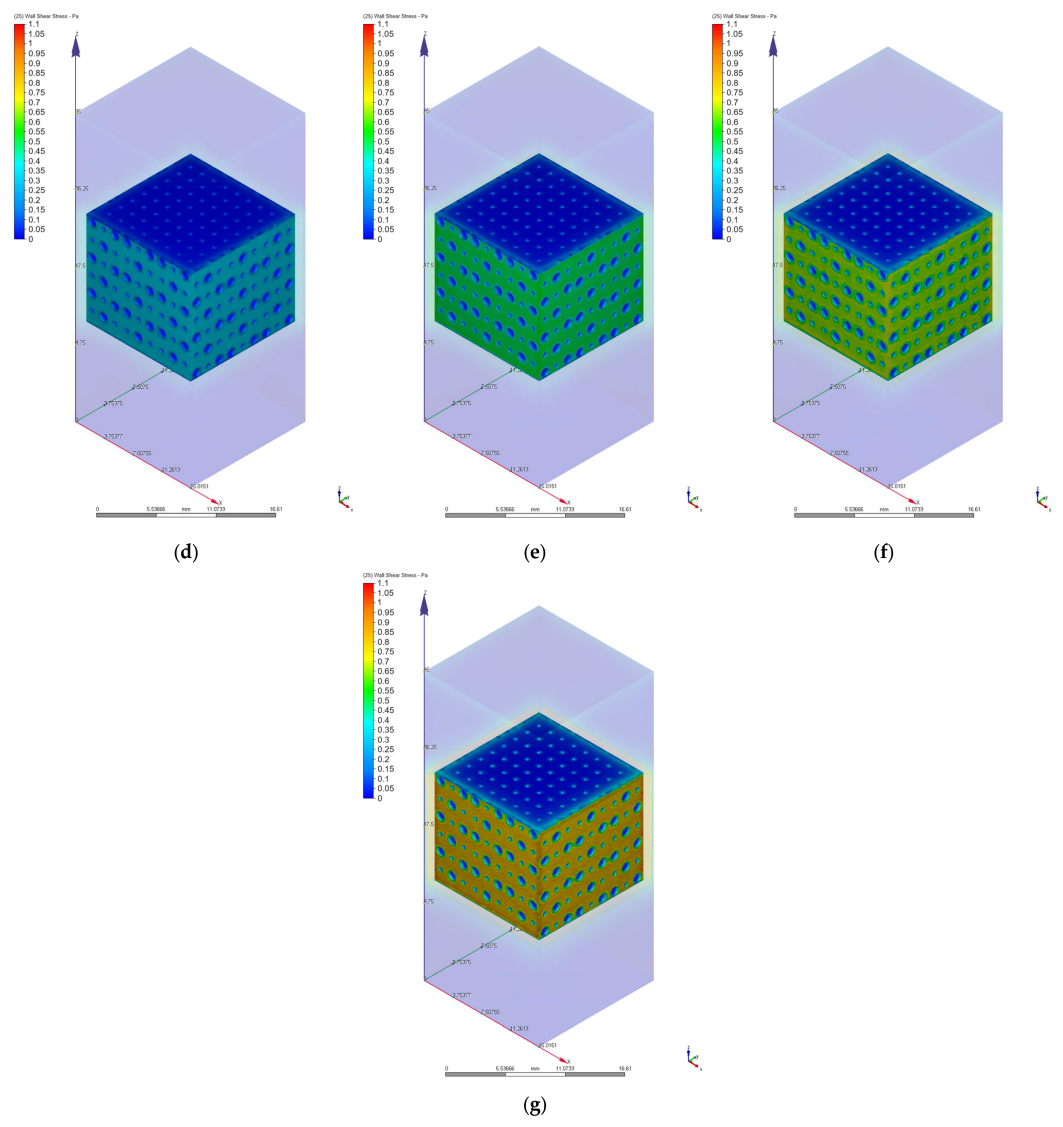
Wall shear stress variation for the PC implant for different values of the inlet velocity set as boundary conditions: (**a**) 1 mm/s; (**b**) 2 mm/s; (**c**) 3 mm/s; (**d**) 4 mm/s; (**e**) 6 mm/s; (**f**) 8 mm/s; (**g**) 10 mm/s.

As we mentioned in Section 2.3, different values of 0.1 mm/s, 1 mm/s, 2 mm/s, 3 mm/s, 4 mm/s, 6 mm/s, 8 mm/s, and 10 mm/s were chosen for the velocity value on the inlet surface during the CFD simulations. Figure 10, Figure 11, Figure 12, Figure 13, Figure 14 and Figure 15 present numerical results obtained for the PP and PC models used for implant permeability calculation based on Equation (3).

In Table 3 are summarized the main numerical results used for implant permeability computation and the Reynolds number obtained in the case of the two CAD models.

**Figure 16:**
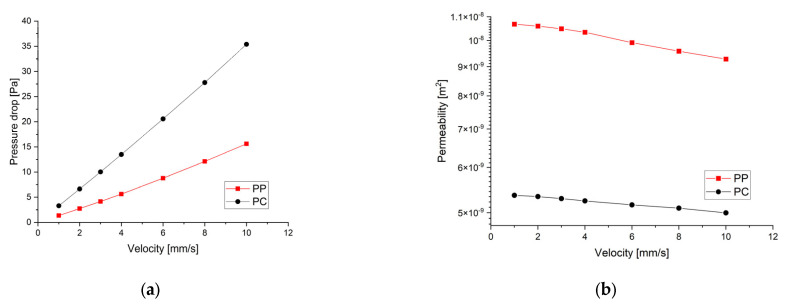
Graphical dependencies obtained after the CFD simulations made for the two implants: (**a**) pressure drop–velocity dependence; (**b**) implant permeability variation as a function of fluid flow velocity.

In Figure 16a is presented the pressure drop dependence determined between the inlet and outlet surfaces of the implants as a function of the fluid flow velocity. It can be noticed that the pressure drop varies linearly with the velocity increase (Figure 10, Figure 11, Figure 13 and Figure 14). The highest value of 35.39 Pa was achieved for the PC implant at a velocity of 10 mm/s compared to 15.624 Pa in the case of the PP implant. This difference can be explained based on the fact that the pore dimensions and implant architecture are different. The high value of the pressure drop numerically computed could be attributed to the important fluid dynamic interactions between implant and fluid due to an increased value of the specific area and a reduced porosity of about 38%. In the case of the PP implant, the higher porosity of about 62% and square-shaped pores determine much more reduced interactions between fluid and implant, so as a direct consequence, a lower pressure drop was achieved. It was observed that the Reynolds number had a maximum value of 6.89 for all the imposed fluid velocity values, and the implant permeability can be correctly estimated by Darcy’s law. Figure 16b shows that the implant permeability decreases when the fluid velocity increases, showing a much more pronounced effect of the pore geometry and implant architecture for the low value of the fluid velocity. In addition, we must mention that this observation remains valid for both designed implants. The implant PP has a maximum permeability of 10.67 × 10^−9^ m^2^ at a velocity of 1 mm/s compared with 5.363 × 10^−9^ m^2^ obtained for the PC implant. Regarding the wall shear stress values for the PP implant (Figure 12), it can be noticed that for a velocity *u* = 1 mm/s, the maximum value is about 20 mPa, which can be considered an optimal value for the biological range, ensuring cell viability. As long as the flow velocity increases, the following values for WSS placed on the implant are achieved: 60 mPa (*u* = 2 mm/s), 80 mPa (*u* = 3 mm/s), 100 mPa (*u* = 4 mm/s), 180 mPa (*u* = 6 mm/s), 280 mPa (*u* = 8 mm/s), and 340 mPa (*u* = 10 mm/s). These high values of the maximum WSS are located exclusively around the implant’s exterior walls. Meanwhile, in the interior of the implant the fluid flows can be considered a beneficial medium for cell adhesion and proliferation, that, according to the literature [24,25,26,27,28], occur in the case of a WSS below a 30 mPa limit. In addition, we can estimate that cell adhesion is difficult on the implant’s exterior surface while it is enhanced on the interior. For the PC implant (Figure 15), a similar behavior was evidenced by the CFD simulations, and the following values for WSS measured on the implant exterior surface were detected: 50 mPa (*u* = 1 mm/s), 200 mPa (*u* = 2 mm/s), 300 mPa (*u* = 3 mm/s), 360 mPa (*u* = 4 mm/s), 450 mPa (*u* = 6 mm/s), 700 mPa (*u* = 8 mm/s), and 800 mPa (*u* = 10 mm/s). The same conclusion regarding the increased cell viability on the implant’s inner volume can be foreseen.

**Figure 17:**
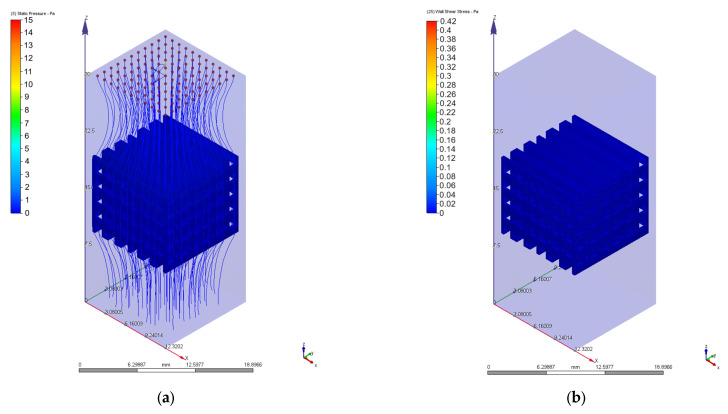

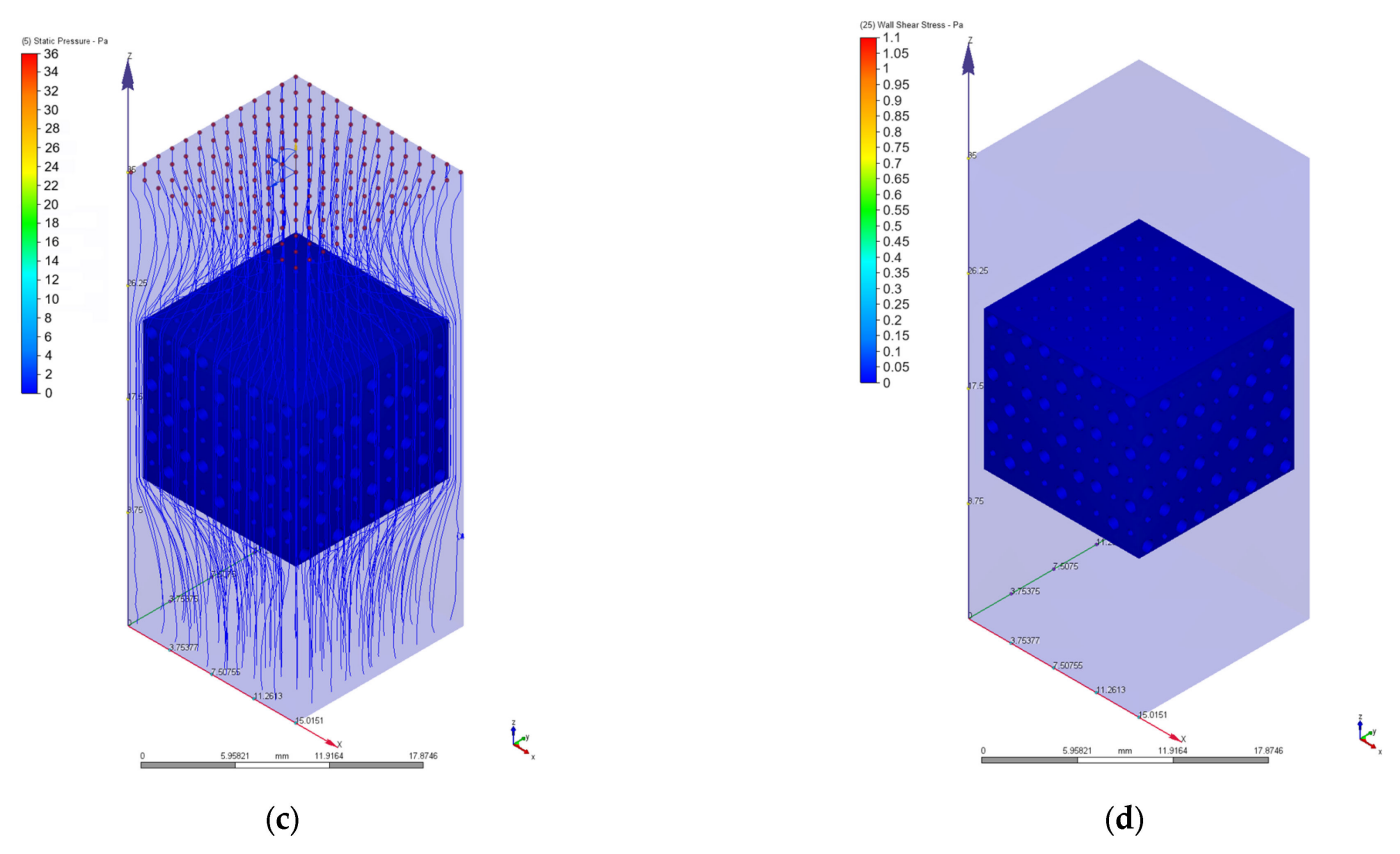
Specific conditions for bioreactors (*u* = 0.1 mm/s): (**a**) pressure drop for the PP implant; (**b**) WSS map for the PP implant; (**c**) pressure drop for the PC implant; (**d**) WSS map for the PC implant.

We tested a very low fluid velocity of 0.1 mm/s and found that below this value specific to bioreactors, the WSS values are much lower than 30 mPa on both implants’ exterior surfaces and interior volumes. It can be concluded that implant cell population should be performed in bioreactor conditions at such reduced values of fluid velocity because values of WSS higher than 60 mPa were not observed (Figure 17).

## 4. Conclusions

Permeability and Young’s modulus of implants dedicated to bone defect treatment must be in accordance with trabecular bone properties (Young’s modulus of 20 GPa and permeability of 5.13 × 10^−9^ m^2^ [18,81,82]). The theoretical values of these physical properties obtained for the two designed implants in conformity with the numerical simulations evidenced that our models are adequate for regenerative implants in bone tissue engineering. Following the WSS analysis, we can conclude that in the case of the low fluid flow velocities met in bioreactors, the cell viability is increased on the inner volume and on the outer surface of the implant. On the other hand, for velocity values close to those of blood flow, which occur in the case of a fracture, one must consider that the cell viability is enhanced on the implant interior and decreased on the exterior surface. It can be recommended that the implant cell population be made in conditions close to those of bioreactors and then implanted at the defect site.

The FEA-CFD simulations permit the mathematical reproduction of linear compression and fluid circulation natural phenomena. The FEA revealed that both implants have mechanical properties close to those of trabecular bone that are adequate for bone defect treatment. It can be noticed that the PC implant has a higher value of Young’s modulus than the PP model, but both values are in the biological range of trabecular bone, helping reconstruct the hard tissue in different parts of the human body.

Current implant design considers that the implant materials have a specific and constant biodegradation rate. Future analysis will be made to validate both implants’ experimental designs for bone defect treatment. In addition, looking to future studies, we want to use non-Newtonian fluids and transient simulations because developing numerical strategies for bone tissue engineering is very important to ensure the success or failure of the designed implants by modifying the mechanical properties and controlling the cell viability.

## Figures and Tables

**Table 1:** Volume and porosity data for the CAD models.

Geometrical Configuration	*V_S_* [mm^3^]	*V_t_* [mm^3^]	*P* [%]
PP	1254.40	470.40	62
PC	2273.13	1396.18	38

**Table 2:** Numerical results obtained after mechanical simulations for the CAD models.

Force [N]	Pressure [MPa]	Minimum von Mises Stress [MPa]		Maximum von Mises Stress [MPa]		Maximum Displacement [mm]		Safety Factor	
		PP	PC	PP	PC	PP	PC	PP	PC
50	0.39	6.49 × 10^−3^	1.54 × 10^−2^	6.222	2.326	5.16 × 10^−4^	1.83 × 10^−4^	42.57	96.46
100	0.79	1.30 × 10^−2^	3.07 × 10^−2^	12.44	4.652	1.03 × 10^−3^	3.66 × 10^−4^	21.28	48.24
200	1.59	2.60 × 10^−2^	6.14 × 10^−2^	24.89	9.303	2.07 × 10^−3^	7.32 × 10^−4^	10.64	24.12
300	2.39	3.89 × 10^−2^	9.21 × 10^−2^	37.33	13.95	3.10 × 10^−3^	1.10 × 10^−3^	7.09	16.08
400	3.18	5.19 × 10^−2^	1.23 × 10^−1^	49.78	18.61	4.13 × 10^−3^	1.47 × 10^−3^	5.32	12.06
500	3.98	6.49 × 10^−2^	1.54 × 10^−1^	62.22	23.26	5.17 × 10^−3^	1.83 × 10^−3^	4.25	9.64
600	4.78	7.79 × 10^−2^	1.84 × 10^−1^	74.66	27.91	6.20 × 10^−3^	2.20 × 10^−3^	3.54	8.04
700	5.58	9.09 × 10^−2^	2.15 × 10^−1^	87.11	32.56	7.23 × 10^−3^	2.56 × 10^−3^	3.04	6.89
800	6.37	1.04 × 10^−2^	2.46 × 10^−1^	99.55	37.21	8.26 × 10^−3^	2.93 × 10^−3^	2.66	6.03
900	7.17	1.17 × 10^−1^	2.76 × 10^−1^	112	41.86	9.30 × 10^−3^	3.30 × 10^−3^	2.36	5.36
1000	7.97	1.30 × 10^−1^	3.07 × 10^−1^	124.4	46.52	1.03 × 10^−2^	3.66 × 10^−2^	2.12	4.82

The pressure was computed as *F*/*A* for each implant, respectively.

**Table 3:** Numerical results obtained after CFD simulations for the two CAD models.

Velocity [mm/s]	Pressure Drops [Pa]		Permeability [m^2^]		Reynolds Number	Maximum Wall Shear Stress [Pa]	
	PP	PC	PP	PC	PP/PC	PP	PC
1	1.35	3.29	1.06 × 10^−8^	5.36 × 10^−9^	0.68	0.04	0.10
2	2.73	6.63	1.06 × 10^−8^	5.33 × 10^−9^	1.37	0.08	0.20
3	4.15	10.02	1.05 × 10^−8^	5.29 × 10^−9^	2.07	0.12	0.31
4	5.61	13.49	1.03 × 10^−8^	5.24 × 10^−9^	2.75	0.16	0.42
6	8.77	20.57	9.91 × 10^−9^	5.16 × 10^−9^	4.14	0.25	0.64
8	12.10	27.78	9.58 × 10^−9^	5.09 × 10^−9^	5.52	0.34	0.87
10	15.62	35.39	9.28 × 10^−9^	4.99 × 10^−9^	6.89	0.43	1.10

## Data Availability

Data are contained within the article.
